# Primary non-communicable disease prevention and communication barriers of deaf sign language users: a qualitative study

**DOI:** 10.1186/s12939-019-0976-4

**Published:** 2019-05-15

**Authors:** Severin Pinilla, Sebastian Walther, Arnd Hofmeister, Soeren Huwendiek

**Affiliations:** 1 University Hospital of Psychiatry, University of Bern, Bern, Switzerland; 2 Institute for Medical Education, University of Bern, Bern, Switzerland; 3 Department of Public Health and Policy, University of Liverpool, Liverpool, UK; 4 Present address: University Hospital of Psychiatry, Bolligenstrasse 111, 60, 3000 Bern, Switzerland

**Keywords:** Deaf, Non-communicable diseases, Diabetes, Sign language, Communication, Prevention

## Abstract

**Background:**

Deaf sign language users have lower health literacy and poorer access to non-communicable disease prevention information as compared to the general population. The aim was to explore disease concepts embedded in signs, primary non-communicable disease prevention behaviour and communication barriers among members of a deaf community.

**Methods:**

A qualitative study with a social constructivist approach was conducted to explore perspectives of deaf sign language users.15 individuals, two with and 13 without history of diabetes were recruited for semi-structured in-depth interviews in sign language at a deaf community center. The interviews were video-recorded, translated and analyzed using thematic content analysis.

**Results:**

Diabetes as one of the main non-communicable diseases is conceptualized differently in the manual component of signs depending on how deaf sign language users construct diabetes pathophysiologically. The disease conceptualization is not represented in the mouthing component. Health information seeking behavior varies among deaf sign language users and depends on their individual spoken and written language literacy. Overcoming communication barriers is key for developing an understanding of diabetes and other non-communicable disease prevention activities.

**Conclusions:**

To develop barrier-free and inclusive non-communicable disease and diabetes prevention strategies for deaf sign language users, health professionals need to pay attention to sign language specific linguistic concepts. More studies are needed to better understand the specific needs of sign language users and effective strategies in health promotion contexts for sign language users.

## Background

The risk of acquiring non-communicable diseases (NCDs) such as diabetes is not equally distributed between and within countries [7, 12]. In fact, the overall lifetime prevalence of diabetes for individuals with low socioeconomic status (SES) was reported at 10.9% and for individuals with a high SES only 4.8% [12]. There is a wealth of data on how low educational status and low health literacy are associated with higher individual diabetes risk [17, 22].

Diabetes is one of the main non-communicable diseases and studies on the global and the national level have shown that the diabetes prevalence is increasing [7]. Its prevalence has increased on the global level from 8.3 to 9.8% in men and from 7.5 to 9.2% in women within 28 years, as well as in Germany from 5.2 to 7.2% within 14 years [7, 12].

Cultural minorities such as deaf sign language users face multiple barriers in terms of health education, socioeconomic status and health information access [18, 33]. Existing research in other fields of health care has shown consistently across countries that deaf sign language users suffer from poor doctor-patient communication [20, 21, 24]. Sign language is key to make relevant information accessible and typically is not part of health care services. To our knowledge no quantitative or qualitative research has been conducted to understand the prevalence of diabetes among deaf sign language users and health information access of deaf sign language users with regards to diabetes.

As a consequence of the unique linguistic structure of sign language, there are differences in terms of health and disease prevention information access in the community of deaf sign language users as compared to the general population [24]. In order to become health literate and actively participate in disease prevention programs these must be comprehensible for deaf sign language users [20, 21]. Studies also show that effective disease prevention for deaf sign language users needs to take several aspects of communication barriers, including pervasive disempowerment resulting from deaf sign language users themselves not acknowledging their own barriers to health information and overprotection through hearing relatives [19–21].

To our knowledge no research has been conducted on the deaf community in the context of specific non-communicable disease or diabetes prevention.

In order to develop effective and inclusive prevention campaigns, a better understanding of disease and language-mode-dependent concepts is necessary. No study was found that explored diabetes specific perceptions of deaf sign language users. Only one quantitative study investigated the knowledge of deaf patients on cardiovascular risk factors and found a significant lower awareness among culturally deaf individuals [24]. Although this study used face-to-face interviews in American Sign Language (ASL), the authors did not comment on their findings with regards to disease concepts embedded in signs.

The goal of the present study was to explore the perceptions of deaf sign language users regarding primary NCD and diabetes prevention, health information access and communication barriers. We aimed at exploring the perspectives of healthy sign language users (and in particular diabetes specific sign language vocabulary) among members of a deaf community. We wanted to identify relevant diabetes sign language vocabulary, emerging recurrent themes of perspectives on communicating with health care professionals of deaf sign language users and strategies to ensure effective patient-centered communication. Answering these questions will allow informing inclusive primary prevention activities, barrier-free population-based health reporting and will help to improve health care services that deliver non-communicable disease prevention activities for deaf sign language users.

## Methods

### Study design

A qualitative study design within a social constructivist approach was used [32]. The first author (SP) conducted a total of 15 video-recorded semi-structured in-depth interviews in German Sign Language (GSL). The interviews were translated into English, transcribed and then analyzed using thematic content analysis [4].

### Study site and participants

All 15 interviewees were members of the deaf community located in Munich, Germany. The term ‘deaf community’ is used here in an informal way and refers to all sign language users who live in the Munich area. The exact number of members and socio-demographic data were not available at the time. A deaf social worker (T.W. personal communication, June 4, 2014) estimated that about 2100 individuals belong to the deaf community in Munich. Members are loosely organized within several different sports and political associations. The largest political and cultural deaf association in the Munich region is the ‘Gehörlosenverband München und Umland e.V.‘(GMU) [11]. Cooperating with the GMU also allowed for recruiting and interviewing at the GMU center.

Important characteristics of all interviewees included predominant use of sign language, deafness before the age of two and willingness to engage in in-depth interviews. Defining criteria of deafness, deafhood and belonging to a deaf community were applied as defined in relevant literature (Petitto, [10, 14, 27]). General descriptive statistics of interviewees are summarized in Table 1.

**Table 1:** Descriptive data of research participants

Pseudonym	Age	Highest educational degree	Onset/reason of deafness	Gender	Diabetes status
A	20	High school degree	Birth/genetic, deaf Coda^b^	F	No diabetes
B	25	High school degree	Birth/unknown	M	No diabetes
C	26	Bachelor degree	Birth/unknown	F	No diabetes
D	31	Bachelor degree	Birth/unknown or genetic, deaf Coda	M	No diabetes
E	34	High school degree	Birth/genetic, deaf Coda	M	No diabetes
F	35	Master degree	Birth/genetic, deaf Coda	M	No diabetes
G	37	High school degree	Birth/unknown	M	No diabetes
H	44	Intermediate school certificate^a^	Birth/genetic, deaf Coda	M	Type 1 diabetes
I	47	Intermediate school certificate	Birth/unknown	M	No diabetes
J	48	Intermediate school certificate	Six months/ infection	F	No diabetes
K	48	Intermediate school certificate	Birth/ infection	M	Type 1 diabetes
L	49	Intermediate school certificate	Two years/infection	F	No diabetes
M	52	Intermediate school certificate	Birth/unknown	M	No diabetes
N	59	Intermediate school certificate	Birth/unknown, deaf Coda	F	No diabetes
O	65	Intermediate school certificate	Birth/unknown	M	No diabetes

^a^Intermediate school certificate, refers to the German ‘Mittlere Reife’, which is usually obtained after ten schooling years. ^b^Child of Deaf Adults (Coda)

### Researchers and reflexivity

We acknowledge that data in this study are co-constructed by interactions with the participants, as are the interpretations and meaning we gave to these data [28, 37]. To provide a rich base for contrasting and interpreting the data as well as to challenge potentially biased views, we brought together a multidisciplinary research team: SP has a background in clinical neurology, psychiatry and medical education. He grew up with both parents being deaf sign language users. As a Coda (Child of deaf adults), SP was exposed to sign language from birth and considers himself having adequate but not full access to Deaf World Knowledge [9, 34]. SW has a background in psychiatry and neurobiology research, AH has a background in public health, disease prevention and qualitative research, SH has a background in pediatrics and medical education.

### Sampling and data collection

To explore the perspectives of members of the deaf community in Munich on diabetes, semi-structured interviews in GSL were conducted by SP. The interviews took place in the GMU center, were video-recorded and saved as QuickTime movie files. SP prepared an interview guide with open-ended questions and adjusted questions based on emerging themes in each interview. Understanding of disease concepts was probed in each interview with ad hoc follow-up questions depending on interviewees’ spontaneously offered signs. Interpretations of disease specific signs were additionally discussed with a certified deaf sign language teacher at the deaf community center. SP interpreted and transcribed each interview to written German. Second, the transcripts were translated to written English and used for thematic content analysis [4]. Each interview was organized and saved as Microsoft Word file.

Both a purposive sampling approach and snowballing was used to recruit participants at the local deaf association center of the GMU and to obtain rich original data from deaf individuals with and without direct diabetes experience [6, 25]. Word-of-mouth recommendations for interviewees were used in order to recruit participants who would have been difficult to reach otherwise, for example deaf individuals with Type-1-diabetes. Gatekeepers such as the president, the vice president, sign language teachers and a social worker of the deaf association were included in the whole project as recommended in the literature [23, 26]. The interviewees were selected either through direct contact of SP with interviewees during the project work at the deaf community center or via recommendations of community members. All contacted community members were invited to participate in the study and there was no coercion to participate.

GSL competence enabled the first author (SP) to conduct the interviews and to build a good rapport with deaf interviewees. Power imbalances were mitigated through establishing safe interview environments by emphasizing the role as student researcher, continuous reflection on the research relationship, and assuring the interviewees of data safety, interviewee privacy and the intention to reduce communication barriers for the deaf community.

### Data analysis

A qualitative data analysis approach [4, 31] was used to identify relevant themes and subthemes from interviewees’ accounts. Individual accounts were explored and contrasted to each other in order to better understand individual and GSL specific diabetes and diabetes prevention related concepts as well as perceptions of communicating with health care professionals [8]. Interpretations of disease specific signs were discussed with a certified deaf sign language teacher at the deaf community center.

## Results

**Fig. 1:**
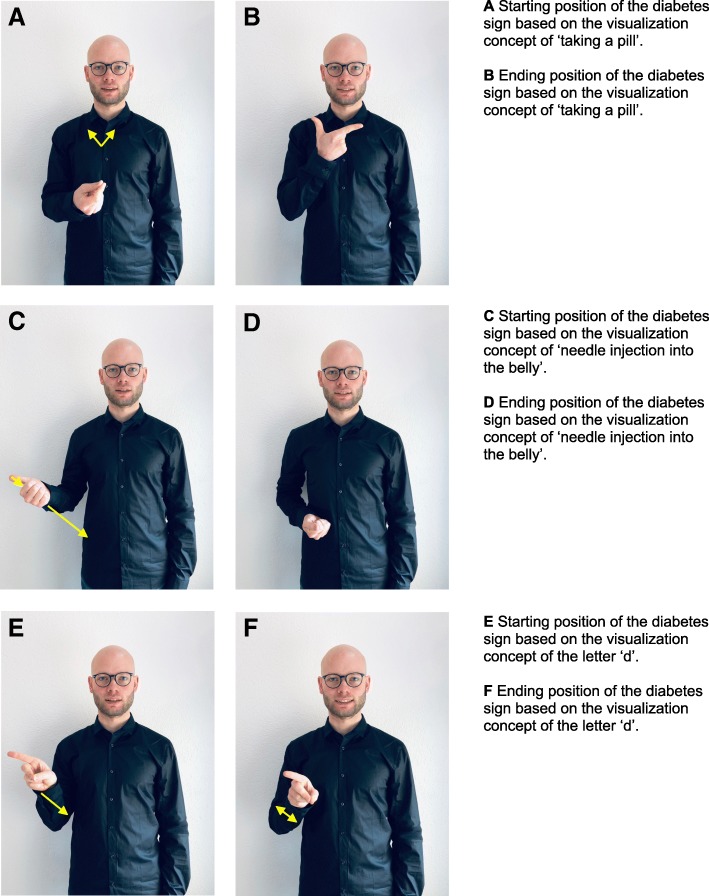
Embedded disease concepts of diabetes in sign language according to manual component. **a** - **f**. Starting and ending positions with indication of movement direction (yellow arrows indicate the direction of movement) for different diabetes signs. All signs are based on the same mouthing element ‘diabetes’

Ten subthemes were identified and grouped into four main themes (Table 2). Emerging diabetes specific German Sign Language (GSL) vocabulary is depicted in Fig. 1 in order to illustrate how different manual components of diabetes signs represent different disease concepts. SP introduced the topic diabetes initially always by fingerspelling the word. In one case diabetes was not known at all to the interviewee. SP therefore offered several signs and basic disease concept explanations before moving on to other questions. Of all 15 interviewees 4 (27%) knew of two different diabetes types, 10 (67%) had only heard of the disease but weren’t able to explain what it is, and one interviewee had never heard of it. Most interviewees used spontaneously the ‘needle sign’ (Fig. 1c and d, *n* = 10 (67%)) and 4 (27%) a sign for sugar disease (not depicted in Fig. 1). The other interviewees either used the ‘shaking d’ (Fig. 1e and f), ‘taking a pill’ (Fig. 1a and b) or differentiated between type 1 and type 2 diabetes (not depicted in Fig. 1). Only one interviewee spontaneously used the full range of diabetes signs.

**Table 2:** Thematic key findings (main theme and associated themes)

1. General diabetes perception	
1.1 Signs for diabetes differ according to the underlying concept of diabetes	
1.2 Diabetes knowledge depends on personal experience and social environment	
1.3 Diabetes is perceived as a private and personal issue	
2. Health information seeking behavior depends on personal health status	
2.1 Learning from a friend or having a disease yourself influences your knowledge	
2.2 The main source for health information is the Internet and different online presentation modalities are used	
3. Learning about general prevention	
3.1 Parents and peers as most important hidden health promoters	
3.2 Acute change in the personal health condition is a trigger to adopt a healthier life style	
4. Persisting communication barriers with health professionals	
4.1 Sign language is the preferred way of communicating and deaf culture should be taken into consideration	
4.2 Get a sign language interpreter	
4.3 Use supportive communication strategies

### Main theme 1: general diabetes perception

The spontaneously expressed concepts of diabetes and reasons for developing diabetes ranged from medical and pathophysiological definitions of diabetes to concepts such as infection through aerosols or simply fate. The depth of thoughts and knowledge on diabetes seemed to be reflected immediately through the range of signs used to describe diabetes (Fig. 1). Each diabetes sign differed with regards to the form and spatial orientation of the signing hand, the position and movement within the signing space, but not with regards to the non-manual parameters mouthing, position of the head and upper body and facial expression.

#### Theme 1.1: signs for diabetes differ according to the underlying concept of diabetes

Different manual parameters were used to represent disease concepts within the sign. Whereas the ‘shaking d’ (Fig. 1e and f) was similarly abstract as the word ‘diabetes’ in spoken language, the ‘insulin needle’-sign (Fig. 1c and d) uses the visualization of ‘using an injection needle in the belly area’ together with mouthing ‘diabetes’ and implied that diabetes is a disease where something needs to be injected in the belly. Depending on how aware a sign language user was of the different types of diabetes, the chosen sign for talking about it was more or less abstract. Using a range of signs for diabetes seemed to be indicative of a more in-depth understanding of the disease concept.

#### Theme 1.2: diabetes knowledge depends on personal experience and social environment

Diabetes was perceived as an acute disease that happens to affect someone and is thus not relevant unless someone got it, as illustrated in the response of Interviewee M after being asked, whether diabetes is important to him: “No (...) not really [...] when I actually have it (...) then of course I would try to find everything that’s out there [...] search the internet (...) try to get all the information from the doctor who diagnosed it (...) you know of course with a sign language translator” (Interviewee M).

For those having experienced type 1 diabetes (T1DM) directly or indirectly they classified diabetes as an acute and unavoidable disease, whereas direct or indirect experiences with type 2 diabetes (T2DM) led to an image of diabetes as a disease, which is related to older age only.

#### Theme 1.3: diabetes is perceived as a private and personal issue

Deaf individuals with diabetes seemed to perceive diabetes in general as a personal and private fate, not as a public health issue. Open discussions within the community of deaf were perceived as difficult: “The deaf I know, who have diabetes (...) I couldn’t ask them detailed questions you know [...] so my knowledge doesn’t go beyond what we said [...] it just doesn’t feel right (...) I mean they have it (...) and I just imagine they wouldn’t want to be asked about that you know (...) I don’t want to be nosy (...)” (Interviewee M).

### Main theme 2: health information seeking behavior depends on personal health status

Interviewees only searched diabetes specific health information if they were either directly affected or if someone in their close social environment had been diagnosed with diabetes. None of the non-affected interviewees had specifically looked for health information on diabetes. Friends and peer groups determine the content and type of exposure to disease specific health information and whether interviewees actively searched for disease specific information.

#### Theme 2.1: learning from a friend or having a disease yourself influences your knowledge

Non-affected deaf interviewees mentioned random and passive exposure to diabetes-related information: “(...) during my time in A. [refers to a university] actually, I had a hearing friend, who had diabetes [needle sign] and had a difficult time managing his blood sugar (...) ah yes, and I remember he had told me that there actually exists type 1 and type 2 diabetes.” (Interviewee E).

Deaf interviewees with T1DM were enrolled in disease management programs, which included health education seminars and still experienced communication barriers: “The next Monday already I had the first session, the seminar actually went for the whole week [...] but you know there were many situations where I felt quite bored, because I didn’t understand anything, [...] and I missed a lot, although I had told everyone that I was deaf [...]” (Interviewee K).

#### Theme 2.2: the main source for health information is the internet and different online presentation modalities are used

Interviewees used the Internet as primary source to search for general and specific health information: “Yes, if I research something then basically always through the internet” (Interviewee C). It emerged from interviewees’ responses that they wanted to have the option to choose between watching a sign language video online or reading text online since this would allow for adapting to individual general literacy levels.

The heterogeneity with regards to reading skills among deaf was mentioned (Interviewee F) as a rationale for providing critical information in sign language as well as in order to reach illiterate deaf individuals.

### Main theme 3: learning about general prevention

The term ‘prevention’ as a public health concept was perceived to be associated with prevention of infectious diseases and only one interviewee mentioned that he actively tried to be physically more active in order to prevent getting diabetes later in his life. The interviewees often had difficulties understanding the Latin loan word for ‘prevention’ (despite fingerspelling), which is used in the public health discourse in Germany. The corresponding sign in GSL is conceptually also used for protection. This double meaning caused confusion during the discussion on health-related behavior.

The willingness or interest to become active in terms of diabetes prevention depended on the proximity to diabetes affected individuals or actually having the disease. Being or feeling healthy served as the most important reference in order to actively think about having to change health related behavior. Although interviewees expressed their interest in learning more about prevention, none of the non-affected interviewees actively searched for general health promotion and prevention information.

#### Theme 3.1: parents and peers as most important hidden health promoters

The parents of interviewees were identified as primary health teachers, who taught what should be perceived as a healthy diet: “I don’t really think actively about it [refers to healthy life style], I mean, I know that I have to eat all different kinds of things, not always the same, I know that, my parents always sort of taught me something about food” (Interviewee C).

Furthermore discussions about healthy lifestyles within the deaf community represent another path of health promotion.: “And then I also started to read a bit about that, and learned that you really shouldn’t add to much sugar to your drinks (...) and also sugar covers up the real taste of things, because it is so strong then (...) so I always try to make people use less sugar (...) you know, people don’t realize how much sugar they add to everything” (Interviewee E).

#### Theme 3.2: acute change in the personal health condition is a trigger to adopt a healthier life style

Interviewees shared that the reason for changing risk behavior resulted from a worsening health condition, and not from trying to prevent the onset of a specific disease or condition.

Whereas interviewees didn’t see any need for action in terms of changing their health behavior because they felt healthy, one interviewee described a perceived conflict between feeling free as an important part of his general health concept and ‘not being free’ if being too concerned about what one should eat and what not: “[laughs] sometimes I eat really bad Bavarian dishes (...) like joint of pork (...) with beer [...] but when I watch others (...) and what they eat (...) like all that bio vegetable stuff (...) they don’t seem to be free (...) like you have to think all the time [...] but it’s really more important to me to feel healthy [...]” (Interviewee M).

### Main theme 4: persisting communication barriers with health professionals

Communication barriers resulting from lack of sign language competence among health professionals and ineffective infrastructure to organize sign language translators emerged in all interviews. Sign language was the preferred way of communicating for all deaf sign language users.

#### Theme 4.1: sign language is the preferred way of communicating and deaf culture should be taken into consideration

Without exception but in varying emphasis the interviewees stated that sign language was the preferred way of communicating with health care professionals: “I mean it is crystal clear, without a translator there is no effective communication between the doctor and me (...) I communicate in sign language and they communicate in spoken language.” (Interviewee H).

Aspects of deaf culture were emphasized: “Some primary care physicians (PCP) maintain this huge distance sort of (...) that doesn’t match the deaf culture, we need more closeness you know (...) and openness in terms of interacting with each other [...] just knowing sign language is not enough for me to choose to go to a PCP (...) the PCP has to understand deaf culture.” (Interviewee E).

#### Theme 4.2: get a sign language interpreter

The identified alternative to a sign language competent health care professional was an official sign language interpreter. However, some interviewees were resistant to using a sign language interpreter but instead expressed to be proud of not needing one. While in cases of perceived severity of a health condition, interviewees expressed that they would always use a sign language interpreter.

In order to overcome privacy issues due to small local deaf communities, a strategy that emerged from the interviews was based on recruiting specific and trusted sign language interpreters, who learned to interpret specifically for a health condition of their client.

#### Theme 4.3: use supportive communication strategies

Interviewees appreciated the efforts made by health care professionals who proactively tried to establish communication and adapted to the deaf way of communicating (Table 3): visualizing as much as possible, taking enough time, maintaining eye contact, writing down notes, speaking clearly or using simple gestures to provide additional visual information: “When I was very active in my sports team, I was injured so many times [...] I had to see an orthopedist very often, and the communication went really well (...) the doctor used a lot of gesticulation when explaining and that helped a lot to understand better, together with lip reading.” (Interviewee C).

**Table 3:** General communication strategies for health professionals working with deaf sign language users

Elements	Explanation
Adequate light	There needs to be enough light so that facial expressions and lips of all individuals present can be seen well
Maintain eye contact	Eye contact should be maintained throughout the conversation to make sure that lip reading is made as easy as possible
Mouthing/Volume	Articulation of words should be as clear as possible (‘speaking as if one was whispering’). Speech should be a bit slower as compared to normal conversation. Lips need to be visible at all times (if possible no surgical mask).
Difficulty of language/ Written information	Explanations should be made with simple words and technical terms should be avoided, since literacy levels of spoken language differ. If necessary, additional written notes should be provided. It should be kept in mind that written information is like a second language for some deaf sign language users.
Using sign language interpreters	Ideally, accredited sign language interpreters should always be present in order to ensure an effective and nuanced communication environment. Interpreters should sit next to the treating physician, so that direct contact with the patient can be maintained.

## Discussion, conclusion and practice implications

### Discussion

#### Concepts of diabetes among sign language users and health information sources

We found that diabetes concepts are implicitly embedded in the signs of sign language. The sign used could be seen as an indicator of both the underlying disease conceptualization and the level of knowledge and health literacy (the more abstract the sign, the higher the health literacy level). Diabetes knowledge as well as health information seeking behavior depends on the personal health experience, the immediate social environment, and the overall literacy level. Furthermore, we found that diabetes was misunderstood as a primarily acute condition. Most deaf sign language users were not aware of the possibility of diabetes prevention by specific measures. Therefore, we suggest that the linguistic structure of sign language needs to be considered when planning and designing education activities for deaf patients to address low health literacy levels [29, 30].

The predominant and preferred source for health information among interviewees was the Internet. Although sign language unanimously was the preferred way of interaction in general, the option to have a sign language translation for every piece of information was not seen as necessary. The emerging preference was an introductory sign language video with particular emphasis on complex health-related information, so that written texts would be easier to understand. This is supported by studies in other health contexts of deaf adults [29, 30] and evidence that the availability of health information online influences how individuals seek information [3].

#### Integration of culture sensitive communication skills training in medical curricula

The findings of this study indicate, that understanding the specific communication culture and structure of sign language is necessary to provide deaf sign language users with adequate access to diabetes related health information. Currently, there is neither qualitative nor quantitative data available on diabetes perceptions of deaf sign language users and further research is needed to develop targeted and effective prevention measures for noncommunicable diseases such as diabetes.

Sign language emerged as the preferred way of communication in all interviews. This finding is in line with previous research in the context of deaf patients and health care [2, 13, 35]. Supportive measures like writing down notes and lip reading might be helpful for superficial conversations, but not sufficient to educate patients about health promotion strategies, healthy life styles or complicated treatment regimens as needed for advanced diabetes patients. Another challenge known from previous studies is the heterogeneity of literacy among sign language users, ranging from complete illiteracy to academic reading and writing skills [14]. We suggest that specific communication skill training to work with sign language users is integrated in medical communication curricula or training curricula for health services staff and supplemented with video and media information toolkits where feasible.

#### Sign language interpreters and health promotion translation

The findings of this study also indicate that deaf sign language users only insist on having a sign language interpreter for acute illnesses. According to the Health Belief Model, acuity of disease and perceived risk are main triggers for preventive behavior [15]. Therefore, deaf patients might not seek access to relevant prevention knowledge with regards to noncommunicable diseases. Learning about preventive behavior of diabetes needs to be part of regular primary care routine visits and sign language interpreters have to be able to adapt their translation to both health literacy levels of deaf patients and to specific disease concepts.

Previous studies with sign language users also support the key role of sign language translators and health information offered in sign language [16]. Removing these communication barriers is necessary to close the health literacy inequity gap between and within countries for the sociocultural minority of deaf sign language users [1, 2, 36]. Despite many countries having implemented legislative frameworks to close the health access inequity gap for people with disabilities, there is a need to develop and provide targeted resources for the overlooked minority of deaf sign language users [5]. Further studies are needed to better understand health literacy and potentially relevant factors such as educational level in deaf sign language users.

#### Limitations

An important limitation of this study is focus on one community in a high-income country. In order to better understand the needs of deaf sign language users in low- and middle-income countries further studies are needed. Additionally, it would be important to explore whether the emerging variations in signs used are also found in other sign languages such as American Sign Language or Spanish Sign Language. In order to also address secondary and tertiary prevention needs with regards to diabetes and other NCDs, perspectives of deaf sign language users with type 2 diabetes need to be interviewed or surveyed. Complementary data from health care professionals working with deaf sign language users would help to adapt existing policies and support health professionals and organizations.

## Conclusion

In order to develop effective diabetes and non-communicable disease prevention strategies for deaf sign language users, health care professionals need to consider sign language specific communication concepts. Deaf culture and health information seeking behavior within deaf communities need to be integrated in wider health promotion activities.

Together with current efforts to make the educational system inclusive for individuals who communicate in sign language, health policy makers should integrate barrier free educational interventions that address prevention activities for non-communicable diseases. Health care professionals need to be made aware of the particular vulnerability of deaf patients to establish safe and effective health care for deaf sign language users.

The qualitative data we present here are limited to a small group of sign language users in a particular context and additional quantitative data as well as health policy analyses are necessary to make robust recommendations for health service improvements. However, to strengthen the rigor of the study the findings were presented, discussed and thereby validated in discussions with an expert panel in the deaf community center in Munich.

Health education interventions for deaf sign language users need to be carefully developed with regards to how specific signs, with implicit disease or health concepts, are used. This applies to disease specific prevention, like diabetes prevention campaigns as well as general life style interventions.

Health care practitioners should provide timely access to sign language interpreters, have adequate knowledge of deaf communication needs and cooperate with sign language interpreters. Health education institutions should provide material in written language and sign language. General communication recommendations for communicating with deaf sign language users in the health care setting are summarized in the results section in Table 3 and are derived from interviewees’ statements on their experiences with communicating with health care professionals and their suggestions to make communication more efficient and effective.

## Abbreviations

ASL: American Sign Language
Coda: Child of deaf adults
GMU: Gehörlosenverband München und Umland e. V [German for: Deaf association of Munich and surroundings]
GSL: German Sign Language
NCDs: Non-communicable diseases
PCP: Primary care physicians
SES: Socioeconomic status
T1DM: Type 1 Diabetes
T2DM: Type 2 Diabetes
